# Lymphoma Associated with Sjögren’s Syndrome

**DOI:** 10.4274/Tjh-2013.0156

**Published:** 2013-12-05

**Authors:** Tuğba Aktan Köşker, Şükran Erten, Esra Erden

**Affiliations:** 1 Atatürk Education and Research Hospital, Internal Medicine, Ankara, Turkey; 2 Atatürk Education and Research Hospital, Department of Rheumatology, Ankara, Turkey; 3 Ankara University Medical Faculty, Department of Pathology, Ankara, Turkey

**Keywords:** Sjogren’s syndrome, non-Hodgkin lymphoma, malignancy

## TO THE EDITOR

A 50-year-old woman visited the outpatient rheumatology department presenting with heel spurs and radiating pain in the legs and the right hip. She had sicca symptoms and morning stiffness lasting for 15 min. 

Her physical examination was normal other than positive FABERE test of the right hip. Routine biochemical tests and complete blood count were normal. Serological tests were positive for anti-nuclear antibodies (+2) with a speckled pattern, and positive for both anti-SS-A/Ro and anti-SS-B/La antibodies. The erythrocyte sedimentation rate and C-reactive protein level were normal. The rheumatoid factor was 17.4 IU/mL (reference range: 0-14 IU/mL). 

The sacroiliac magnetic resonance findings were compatible with trochanteric bursitis. The results of Schirmer’s test were <5 mm for both eyes and a minor salivary gland biopsy showed focal periductal lymphoid aggregate. With these findings, the present case apparently met the criteria for the diagnosis of Sjögren’s Syndrome (SS) [1]. The patient was treated with hydroxychloroquine and artificial tear drops. 

Eighteen months later, the patient was admitted with painless swelling of the parotid glands. There was generalized peripheral lymphadenopathy in the neck, axillary, and inguinal regions. 

**Figure 1:**
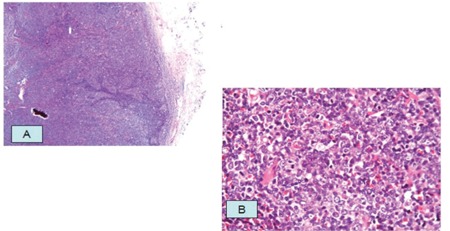
a) Tumor tissue deforming the structure, of lymph nodes and infiltrating extranodal structures b) Tumor cells with large pleomorphic nucleus, apparent nucleolus, and frequent mitosis

Excisional biopsies were obtained from the right inguinal lymph node, which revealed a high-grade, B-cell non-Hodgkin lymphoma (NHL). The tumor tissue was composed of atypical round cells with pleomorphic vesicular nuclei undergoing frequent mitosis and eosinophilic cytoplasm (Figure 1). Immunohistochemically, neoplastic cells were positive for CD20, and the Ki-67 proliferation index was around 70%.

She received 3 cycles of rituximab plus cyclophosphamide, epirubicin, vincristine, and prednisolone. Because of cardiotoxicity, she received 5 cycles of rituximab, cyclophosphamide, and Oncovin.

After 2 months, her symptoms were resolved and her CT scan revealed a remarkable decrease in the generalized swelling of the lymph nodes. Five months later, a PET scan showed no findings consistent with recurrence or involvement of the primary malignancy, and these findings were consistent with remission.

SS is a common autoimmune disorder characterized by the degeneration of exocrine glands, clinically presenting as eye and mouth dryness [2]. Patients with SS have high levels of immunoglobulin, anti-Ro/SSA, and anti-La/SSB. SS is seen in all age groups, but it is more common in the fourth and fifth decades of life [3]. We report a case of NHL associated with SS presenting with enlargement of the parotid gland and generalized peripheral lymphadenopathy.

The incidence of NHL in patients with SS has been reported as 5%. The risk of NHL development in patients with SS is approximately 40-44 times greater than that in the general population [2]. Lymphomas that appear during the course of SS are usually localized extranodal low-grade B-cell NHL and the major histopathological type is mucosa-associated lymphoid tissue lymphoma [4].

The clinical and laboratory signs of lymphoma are based on etiology. The reported extranodal sites are mostly the salivary glands, followed by the stomach, nasopharynx, skin, lung, lachrymal gland, liver, and bones. In this disease, although rare, central nervous system involvement can also be seen [5].

In conclusion, we encountered a rare case of SS complicated with NHL. We recommend that patients with SS be carefully evaluated for occult malignancy and in particular for NHL. 

## CONFLICT OF INTEREST STATEMENT

The authors of this paper have no conflicts of interest, including specific financial interests, relationships, and/ or affiliations relevant to the subject matter or materials included.
